# Comparative efficacy and toxicity of two versus three induction chemotherapy cycles in locally advanced nasopharyngeal carcinoma: a propensity score matching analysis

**DOI:** 10.1186/s12885-026-16655-2

**Published:** 2026-07-31

**Authors:** Renxian Xie, Xiaoluan Lin, Qiufeng Li, Junqing Pan, Keyan Xie, Jianzhou Chen, Ying Zhang, Chuangzhen Chen, Yizhou Zhan

**Affiliations:** 1 Department of Radiation Oncology, Cancer Hospital of Shantou University Medical College, 7 Raoping Road, Shantou, Guangdong 515031 P.R. China; 2 Shantou University Medical College, Shantou, P.R. China

**Keywords:** Nasopharyngeal carcinoma, Induction chemotherapy, Concurrent chemoradiotherapy, Treatment cycles, Propensity score matching, Acute toxicity

## Abstract

**Background:**

For patients with locally advanced nasopharyngeal carcinoma (LA-NPC), the optimal number of cycles of induction chemotherapy (IC) remains uncertain. This study aimed to assess the efficiency and toxicity of different cycles of IC in LA-NPC patients.

**Methods:**

This retrospective study included 210 patients with LA-NPC and compared clinical outcomes and toxicity in two groups: 129 patients received two cycles of IC plus concurrent chemoradiotherapy (CCRT), and 81 patients received three cycles of IC plus CCRT. Propensity score matching was employed to mitigate selection bias and enhance the comparability of the two cohorts. Survival analysis was conducted using Kaplan-Meier curves, log-rank tests, and Cox proportional hazards regression analyses.

**Results:**

Propensity score matching identified 81 matched pairs of patients. No significant differences were observed between the two-cycle and three-cycle groups regarding 5-year overall survival (OS) rates (88.1% vs. 80.4%, *p* = 0.21), 5-year locoregional relapse-free survival (LRRFS) rates (91.8% vs. 94.7%, *p* = 0.51), 5-year distant metastasis-free survival (DMFS) rates (80.9% vs. 84.8%, *p* = 0.55), or 5-year progression-free survival (PFS) rates (73.4% vs. 79.7%, *p* = 0.4). However, the three-cycle group had a greater incidence of grade 3/4 treatment-related acute toxicity, with the difference being significant during CCRT but not IC. Multivariate analysis identified stage IVa as an independent prognostic factor for OS, while LDH levels ≥ 245 U/L were independent predictors of poorer DMFS and PFS.

**Conclusion:**

For patients with LA-NPC, three cycles of IC did not significantly improve patients’ survival compared to two cycles of IC but rather increased the incidence of toxicity.

**Supplementary Information:**

The online version contains supplementary material available at https://doi.org/10.1186/s12885-026-16655-2.

## Introduction

Nasopharyngeal carcinoma (NPC) is a malignant tumor arising from nasopharyngeal epithelial cells, closely linked to Epstein-Barr virus (EBV) [1, 2]. NPC is more prevalent in certain geographic regions, particularly in Southeast Asia, and is known for its unique clinical and biological characteristics, including a high radiosensitivity [3]. Because of its deep-seated anatomic position and high radiosensitivity, radiotherapy is fundamental to the treatment of nasopharyngeal carcinoma, especially for early localized lesions. Unfortunately, the majority of patients with NPC have localized infiltration or metastases at the time of diagnosis [4]. It is now recommended that concurrent chemoradiotherapy (CCRT) be used for patients with locally advanced nasopharyngeal carcinoma (LA-NPC), with the aim of minimizing toxic side effects while maximizing local lesion control, thereby improving treatment outcomes [5–9]. However, LA-NPC patients exhibit unsatisfactory local and regional control, with about 30% developing disease progression after treatment [10]. Furthermore, patients are subjected to significant treatment-related toxicities, and there is a need to investigate improved therapeutic options to enhance patient survival while lowering treatment-related toxicities.

The addition of induction chemotherapy (IC) to CCRT in the treatment regimen of LA-NPC aims to improve the efficacy of tumor treatment. The aim of this strategy is to reduce the tumor load before starting CCRT, thereby reducing the size of the radiotherapy target area. This approach can reduce the incidence and severity of radiotherapy-related side effects, thereby improving the overall tolerability and quality of life of patients undergoing CCRT [11, 12]. In addition, IC can reduce tumor staging and improve radiosensitivity of residual tumors by reducing tumor volume [12, 13]. Several studies have explored the efficacy and safety of this combined approach, highlighting its potential benefits in enhancing overall survival and progression-free survival rates [14–16].

However, the optimal number of IC cycles remains controversial, largely due to the lack of consensus based on robust evidence. Some studies suggest that two cycles of IC are sufficient to achieve a significant reduction in tumor burden without excessive toxicity [17–19]. On the other hand, some scholars believe that three cycles of IC may better eradicate potential micrometastases in LA-NPC patients, resulting in better local control and survival outcomes. And several clinical trials have used three cycles of IC as a treatment regimen for patients [11, 13, 20]. Unfortunately, 3% to 25% of patients fail to complete three IC cycles due to treatment-related toxicity and costs [21–23]. Therefore, further studies to determine the optimal number of IC cycles are necessary because the duration of IC affects the overall treatment schedule and has a potential impact on the timing of radiotherapy, which is known to be a critical factor affecting outcomes [24].

In this study, we aimed to evaluate the comparative efficacy and toxicity of two versus three cycles of IC in LA-NPC patients. We conducted a propensity score matching (PSM) analysis to minimize selection bias and ensure that any observed differences in outcomes were attributable to the number of IC cycles rather than confounding factors. Our findings will provide additional evidence to determine the optimal treatment modality for LA-NPC. The results of this study may significantly impact clinical decision-making for LA-NPC patients and inform the design of future trials aimed at optimizing treatment strategies and improving survival rates for patients with this aggressive cancer.

## Materials and methods

### Data source

The original data are openly accessible on the internet, and the authors have given their permission for other researchers to use them (https://figshare.com/articles/dataset/Clinic_value_of_nedaplatin_in_nasopharyngeal_carcinoma/12600458), which was accessed on October 18th, 2024 [25]. This retrospective cohort study utilized the baseline characteristics from the dataset, which included the following variables: age, gender, histology, T stage, N stage, clinical stage, IC cycle, platinum dose in CCRT, Body Mass Index (BMI), smoking status, and lactate dehydrogenase (LDH) levels. The study adhered to the Declaration of Helsinki.

### Patient population

A retrospective analysis of patients treated at Guangzhou Medical University’s Affiliated Cancer Hospital & Institute with locoregional advanced NPC from June 2012 to June 2017 was conducted. Senior doctors restaged the clinical stage using the 8th edition UICC/AJCC Classification [26], while the pathological type was identified based on the WHO histological classification of NPC. The Eastern Cooperative Oncology Group (ECOG) standard was used to confirm performance status.

Participants eligible for the study were aged 18 to 70 years, had a pathological diagnosis of WHO type II or III NPC, were classified as clinical stage III or IVa, had an ECOG performance status of 0 or 1, and received 2 or 3 cycles of IC. As exclusion criteria, participants with a history of other malignancies, incomplete clinical data, or severe heart, liver, kidney, or lung disease were excluded.

The IC + CCRT treatment was performed on 269 NPC patients between June 2012 and June 2017. Based on inclusion and exclusion criteria, 210 patients were collected from the two groups: 129 in the IC = 2 group and 81 in the IC = 3 group.

### Treatment procedures

Each patient received simultaneous intensity-modulated radiotherapy (IMRT) in the supine position while wearing a thermoplastic mask that covered their head and neck. From the vertex to 2 cm below the clavicle, a Computed Tomography (CT) scan was performed at 3 mm per slice using a CT simulator.

Using the Pinnacle 9.10 (Amsterdam, Netherlands) treatment planning system, target volumes were determined according to the institutional treatment protocol of the Sun Yat-sen University Cancer Center [27]. The target volumes were defined as follows: The primary gross tumor volume (GTVnx) and the involved lymph nodes (GTVnd) included all known gross disease as determined by the imaging, clinical, and endoscopic findings. For the clinical target volume 1 (CTV1), a margin of 0.5–1 cm was added manually to the GTVnx. CTV2 was defined as CTV1 plus a 0.5–1 cm margin, including the bilateral uninvolved regional nodes (retro- and parapharyngeal nodes and cervical nodes at levels II, III, and the upper portion of level V limited to the inferior body of the cricoid bone). PTVnx, PTVnd, PTV1, and PTV2 were generated by adding a 0.5 cm margin to GTVnx, GTVnd, CTV1, and CTV2, respectively. Care was taken to ensure at least a 5-mm gap was present between the PTVs and the skin. The contoured critical structures included the brainstem, chiasm, optic nerves, spinal cord, temporal lobes, eyes, lens, parotid glands, oral cavity, larynx, mandible, and temporomandibular joints.

Gross tumor volumes were assessed using CT or MRI imaging, physical examination, and nasal endoscopy. The administered dose for the GTVnx and GTVnd ranged from 68 to 70 Gy. The CTV1 received a dose of 60–62 Gy, while the CTV2 received 54–56 Gy. Treatment was administered to all patients in 30–33 fractions, with one fraction per day, five days a week.

Patients in the IC = 2 group received two cycles of IC every three weeks, consisting of docetaxel (60–75 mg/m², day 1), cisplatin or nedaplatin (60–75 mg/m², day 1), and 5-fluorouracil (500–600 mg/m² per day, days 1–5). During radiotherapy, cisplatin or nedaplatin was given at a dose of 80 mg/m² every three weeks or 30 mg/m² every week as concurrent chemotherapy. In contrast, the only difference in treatment between the two groups was the number of cycles of IC they received.

### Follow-up

Following the completion of treatment, the patient underwent follow-up evaluations at three-month intervals during the first two years, at six-month intervals during years three to five, and annually thereafter. During these follow-up visits, comprehensive assessments were conducted, including physical examinations, chest radiography, abdominal ultrasound, electronic nasopharyngoscopy, and magnetic resonance imaging (MRI) of the head and neck. Toxicities were assessed using the Common Terminology Criteria for Adverse Events (CTCAE, version 4.0), and follow-up duration was measured from diagnosis to the last follow-up or death.

Overall survival (OS) was assessed by calculating the interval from the initial diagnosis to either the final follow-up appointment or the recorded date of death. Progression-free survival (PFS), distant metastasis-free survival (DMFS), and locoregional relapse-free survival (LRRFS) were defined as the duration from diagnosis to the first occurrence of disease progression, the onset of distant metastasis, or the incidence of locoregional relapse, respectively.

### Propensity score matching

The propensity score matching (PSM) method is extensively employed in observational studies to reduce selection bias. In this study, PSM was used to match patients from the IC = 2 and IC = 3 groups in a 1:1 optimal pairing, ensuring balance in baseline covariates including age, sex, histology, clinical stage, CCRT platinum dose, BMI, smoking status, and LDH levels.

### Statistical analysis

Differences in clinical characteristics between the two groups were evaluated using a Mann-Whitney U test and a two-tailed Student’s t-test. Kaplan-Meier curves, log-rank tests, and Cox proportional hazards regression were utilized for survival analysis. To compare the incidence of complications between the two groups, chi-squared tests or Fisher’s exact tests were performed. A p-value < 0.05 was considered statistically significant. Statistics were conducted using Free Statistics (version 1.9) and R software.

## Results

### Patient characteristics

**Fig. 1:**
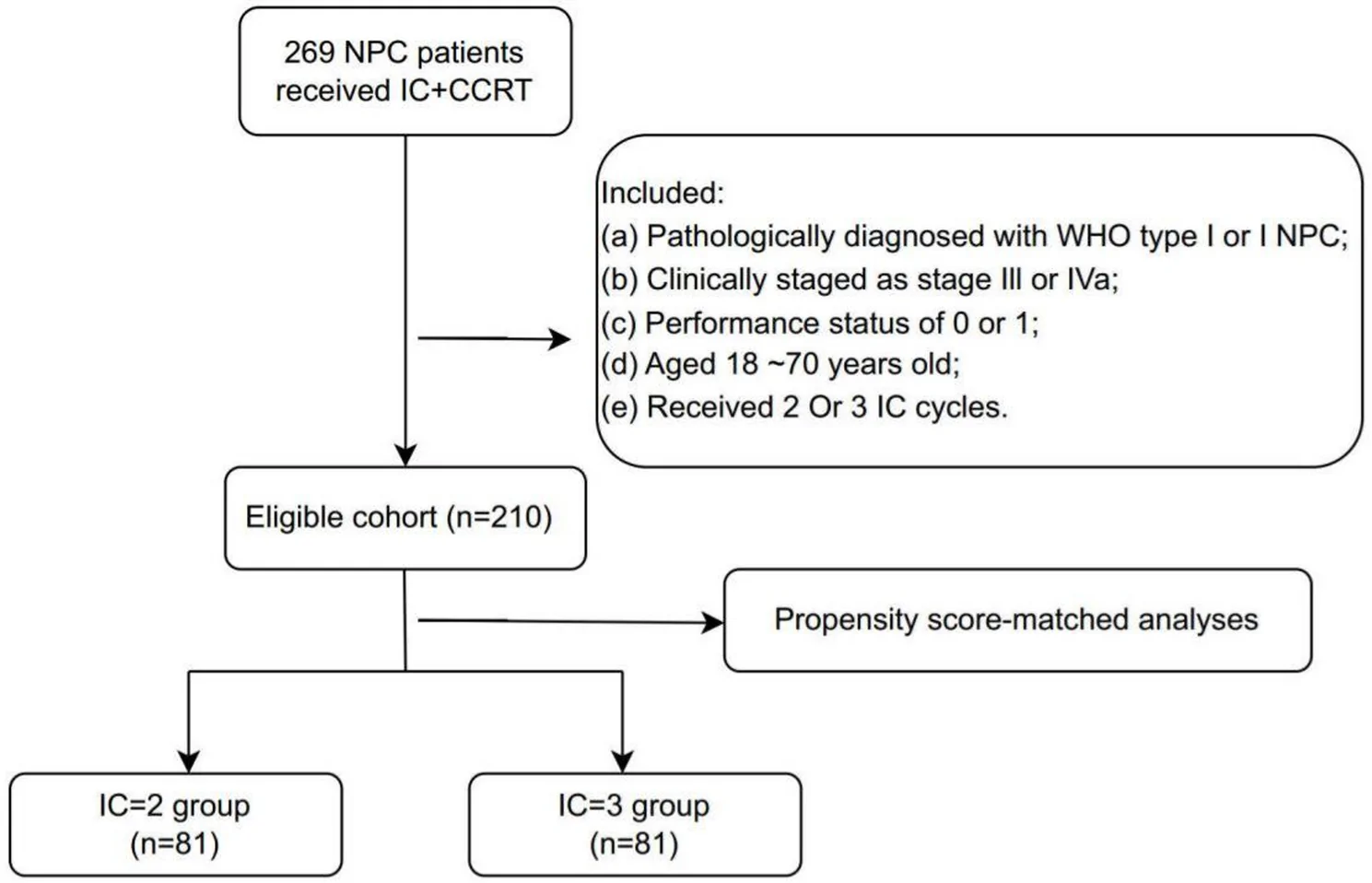
Flow-chart of the patient selection procedure

The flowchart in Fig. 1 shows the process of selecting patients after carefully considering the inclusion and exclusion criteria. 129 patients were placed in the IC = 2 group, and 81 in the IC = 3 group. Table 1 shows the clinical and pathological characteristics of the IC = 2 and IC = 3 groups before and after PSM. Before PSM, more patients in the IC = 3 group were stage N2-3 patients, and more received low-dose cisplatin compared with the IC = 2 group. A total of 81 patients were included in each group following the matching of age, gender, histology, clinical stage, platinum dose of CCRT, BMI, smoking, and LDH levels.

**Table 1:** Patient demographics and baseline characteristics

Characteristics	Before matching			After matching		
	IC = 2 group, *N* = 129	IC = 3 group, *N* = 81	*p*-value	IC = 2 group, *N* = 81	IC = 3 group, *N* = 81	*p*-value
Age (Mean ± SD)	46 ± 11	47 ± 11	0.335	47 ± 10	47 ± 11	0.936
Gender			0.920			0.838
Male, *n* (%)	106 (82%)	67 (83%)		66 (81%)	67 (83%)	
Female, *n* (%)	23 (18%)	14 (17%)		15 (19%)	14 (17%)	
Histology			0.487			> 0.999
WHO II	7 (5%)	2 (2%)		2 (2%)	2 (2%)	
WHO III	122 (95%)	79 (98%)		79 (98%)	79 (98%)	
T stage			0.408			0.603
T1-2	42 (33%)	22 (27%)		25 (31%)	22 (27%)	
T3-4	87 (67%)	59 (73%)		56 (69%)	59 (73%)	
N stage			0.011*			0.527
N0-1	39 (30%)	12 (15%)		15 (19%)	12 (15%)	
N2-3	90 (70%)	69 (85%)		66 (81%)	69 (85%)	
Clinica stage			0.061			0.875
III	76 (59%)	37 (46%)		38 (47%)	37 (46%)	
IVa	53 (41%)	44 (54%)		43 (53%)	44 (54%)	
Smoking			0.056			0.427
No	87 (67%)	44 (54%)		49 (60%)	44 (54%)	
Yes	42 (33%)	37 (46%)		32 (40%)	37 (46%)	
BMI			0.053			> 0.999
< 18.5	12 (9%)	2 (2%)		1 (1%)	2 (2%)	
≥ 18.5	117 (91%)	79 (98%)		80 (99%)	79 (98%)	
LDH level (U/L)			0.552			0.822
< 245	115 (89%)	70 (86%)		69 (85%)	70 (86%)	
≥ 245	14 (11%)	11 (14%)		12 (15%)	11 (14%)	
Platinum dose of CCRT (mg/m^2^)			0.002*			0.149
< 200	56 (43%)	53 (65%)		44 (54%)	53 (65%)	
≥ 200	73 (57%)	28 (35%)		37 (46%)	28 (35%)	
Chemotherapy regimen			0.347			0.752
TNF + P	52 (40%)	38 (47%)		36 (44%)	38 (47%)	
TNF + N	77 (60%)	43 (53%)		45 (56%)	43 (53%)	

*IC* induction chemotherapy, *CCRT* concurrent chemoradiotherapy, *BMI* body mass index, *LDH* lactate dehydrogenase, *TPF + P* docetaxel plus nedaplatin and 5-fluorouracil followed by nedaplatin-based CCRT, *TNF + N* docetaxel plus cisplatin and 5-fluorouracil followed by cisplatin-based CCRT, *PSM* propensity score-matched

### Long-term efficacy

**Fig. 2:**
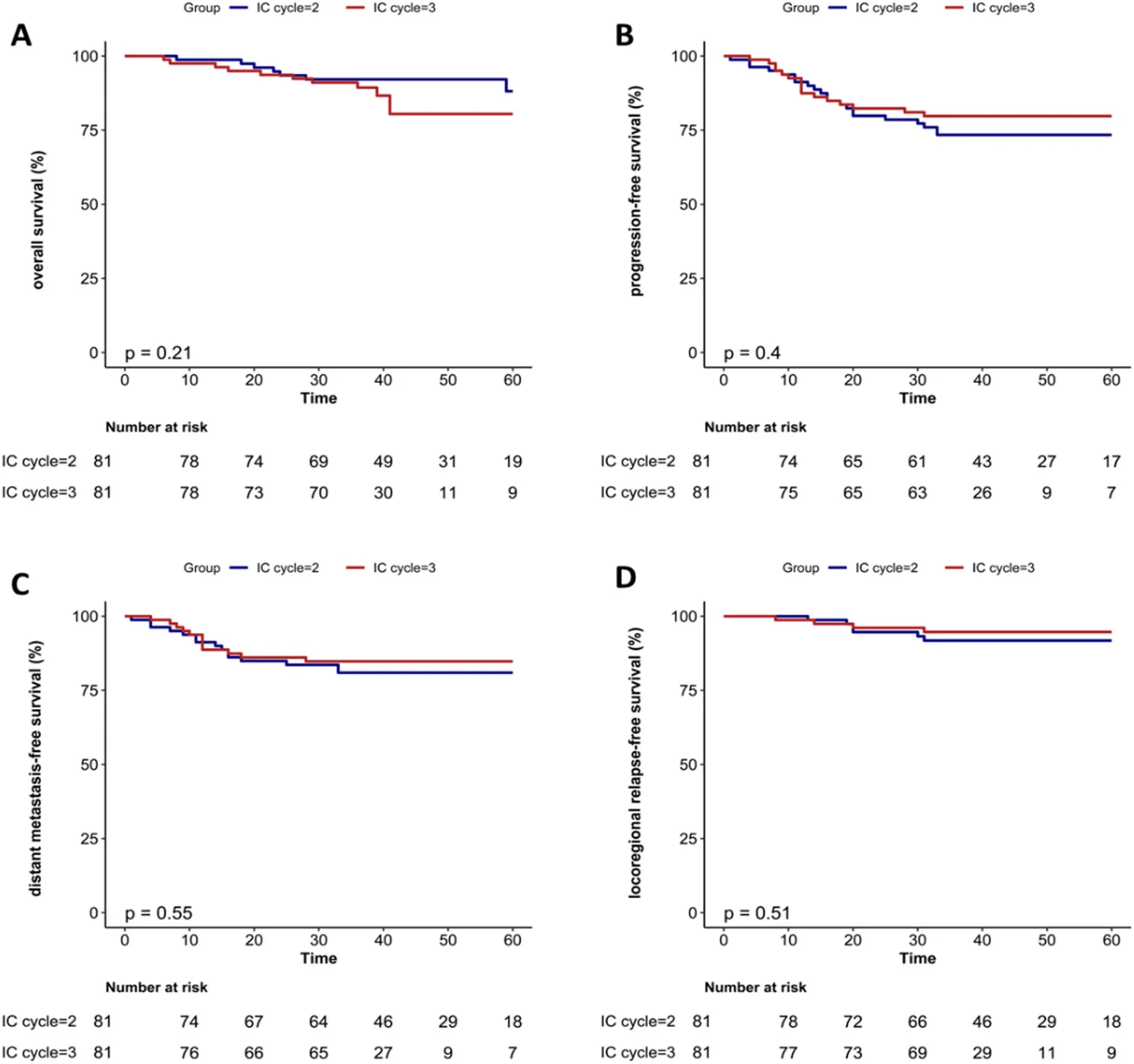
Kaplan-Meier survival curves of the IC = 2 group and IC = 3 group in patients after matching. **A** Overall survival; **B** Progression-free survival; **C** Distant metastasis-free survival; **D** Locoregional-free survival

In this study, the median duration of follow-up was 39 months, with a range from 6 to 94 months. The Kaplan-Meier survival analysis revealed no significant differences in 5-year survival outcomes between the IC = 2 and IC = 3 groups in terms of overall survival (OS) (88.1% vs. 80.4%, *p* = 0.21; Fig. 2A), progression-free survival (PFS) (73.4% vs. 79.7%, *p* = 0.4; Fig. 2B), distant metastasis-free survival (DMFS) (80.9% vs. 84.8%, *p* = 0.55; Fig. 2C), and locoregional relapse-free survival (LRRFS) (91.8% vs. 94.7%, *p* = 0.51; Fig. 2D). Furthermore, after PSM, multivariate survival analysis was conducted, incorporating variables identified by univariate Cox analysis (*p* < 0.05) and clinical expertise into the multivariate Cox analysis. Stage IVa was identified as an independent prognostic factor in the multivariate analysis of clinicopathologic characteristics for OS. In contrast, stage IVa and LDH levels > 245 U/L were independent predictors of adverse outcomes for DMFS and PFS. Table 2 shows the multivariate analyses of the 5-year OS, PFS, DMFS, and LRRFS rates based on Cox proportional hazards regression models after PSM.

**Table 2:** Multivariate analyses of the OS, PFS, DMFS and LRRFS by Cox proportional hazards regression models after PSM

	OS		PFS		DMFS		LRRFS	
	aHR(95% CI)	*p*-value	aHR(95%CI)	*p*-value	aHR(95%CI)	*p*-value	aHR(95%CI)	*p*-value
Age (< 47 vs. ≥ 47)	N/A	N/A	0.66 (0.34 ~ 1.27)	0.211	0.64 (0.3 ~ 1.4)	0.266	N/A	N/A
Gender (Male vs. Female)	N/A	N/A	0.42 (0.13 ~ 1.4)	0.158	N/A	N/A	N/A	N/A
T stage (T1-2 vs. T3-4)	N/A	N/A	N/A	N/A	N/A	N/A	3.89 (0.49 ~ 30.88)	0.199
Clinical stage (III vs. IVa)	6.59 (1.51 ~ 28.85)	0.012*	2.16 (1.05 ~ 4.42)	0.036*	2.58 (1.08 ~ 6.15)	0.033*	N/A	N/A
LDH (< 245 vs. ≥ 245)	2.56 (0.92 ~ 7.17)	0.073	3.42 (1.65 ~ 7.09)	0.001*	3.74 (1.62 ~ 8.6)	0.002*	N/A	N/A
IC cycle (2 vs. 3)	1.83 (0.71 ~ 4.75)	0.212	0.68 (0.35 ~ 1.33)	0.256	0.71 (0.32 ~ 1.55)	0.384	0.68 (0.19 ~ 2.4)	0.544
Platinum dose (< 200 vs. ≥ 200)	1.31 (0.5 ~ 3.45)	0.583	1.3 (0.66 ~ 2.57)	0.445	1.24 (0.57 ~ 2.72)	0.588	2.85 (0.8 ~ 10.2)	0.107
Chemotherapy regimen (TNF + P vs. TNF + N)	N/A	N/A	N/A	N/A	N/A	N/A	0.34 (0.09 ~ 1.32)	0.119

*PSM* propensity score-matched, *OS* overall survival, *PFS* progression-free survival, *DMFS* distant metastasis-free survival, *LRRFS* locoregional relapse-free survival, *aHR* adjust hazard ratio, *TPF + P* docetaxel plus nedaplatin and 5-fluorouracil followed by nedaplatin-based CCRT, *TNF + N* docetaxel plus cisplatin and 5-fluorouracil followed by cisplatin-based CCRT, *N/A* not applicable

### Safety

Table 3 details the occurrence of acute toxicity grades 3/4 in both groups. In terms of non-hematologic toxicities, grade 3/4 vomiting was significantly more prevalent in the IC = 3 group compared to the IC = 2 group (14.8% vs. 4.9%, *P* = 0.035). There were no significant differences between the two groups in terms of weight loss, hypoalbuminemia, or impairment of liver and kidney function. As far as hematologic toxicities are concerned, the IC = 3 group showed a significantly higher rate of grade 3/4 thrombocytopenia (21.1% vs. 4.9%, *P* = 0.002) and a significantly higher rate of anemia (16.0% vs. 6.2%, *P* = 0.046). In contrast, leukopenia and neutropenia incidences were not significantly different between the two groups.

**Table 3:** Incidence of grade 3 acute toxicities during treatment in patients after PSM

Acute Toxicities	During treatment			During IC			During CCRT		
	IC = 2 group, *N* = 81	IC = 3 group, *N* = 81	*p*-value	IC = 2 group, *N* = 81	IC = 3 group, *N* = 81	*p*-value	IC = 2 group, *N* = 81	IC = 3 group, *N* = 81	*p*-value
Haematologic									
Leucopenia	32 (39.5)	27 (33.3)	0.414	7 (8.6)	8 (9.9)	0.786	32 (39.5)	23 (28.4)	0.135
Neutropenia	29 (35.8)	24 (29.6)	0.402	13 (16)	17 (21)	0.418	29 (35.8)	16 (19.8)	0.023*
Anaemia	5 (6.2)	13 (16)	0.046*	1 (1.2)	1 (1.2)	1	5 (6.2)	14 (17.3)	0.028*
Thrombocytopenia	4 (4.9)	17 (21)	0.002*	1 (1.2)	0 (0)	1	5 (6.2)	12 (14.8)	0.073
Non-Haematologic									
Vomiting	4 (4.9)	12 (14.8)	0.035*	3 (3.7)	8 (9.9)	0.118	5 (6.2)	11 (13.6)	0.114
Weight loss	2 (2.5)	2 (2.5)	1	1 (1.2)	2 (2.5)	1	2 (2.5)	2 (2.5)	1
Hypoalbuminemia	0 (0)	0 (0)	1	0 (0)	0 (0)	1	0 (0)	0 (0)	1
Liver/Kidney damage	1 (1.2)	0 (0)	1	1 (1.2)	0 (0)	1	1 (1.2)	0 (0)	1

*PSM* propensity score-matched, *IC* induction chemotherapy, *CCRT* concurrent chemoradiotherapy, *N/A* not applicable

A detailed analysis was performed to assess the impact of IC or CCRT on toxicity between the two groups (Table 3). During CCRT, but not during IC, the IC = 3 group experienced a higher incidence of treatment-related severe acute toxicity (including neutropenia and anemia). Of note, all patients in both the IC = 2 and IC = 3 groups successfully completed the planned concurrent chemoradiotherapy, including the full course of IMRT and at least two cycles of concurrent platinum-based chemotherapy. As this was a retrospective study, patients unable to complete the prescribed radiotherapy or concurrent chemotherapy were excluded at enrollment; consequently, the overall CCRT completion rate was 100% in both groups. 

### Subgroup analysis

Multivariate analyses indicated that patients with advanced clinical stages or elevated LDH levels had a higher likelihood of treatment failure. As post hoc exploratory analyses, we conducted subgroup analyses to evaluate the impact of various IC cycles on patients with different risk stratifications. No formal interaction tests were performed to assess whether the treatment effect of IC cycles differed across subgroups; therefore, these analyses are hypothesis‑generating and should be interpreted with caution. Subgroup analyses revealed no significant survival differences between the two and three cycles of IC for patients stratified by clinical stage and LDH level. Table 4 presents subgroup analyses of survival outcomes.

**Table 4:** Subgroup analysis

Characteristic	OS (%)			PFS (%)			DMFS (%)			LRRFS (%)		
	IC = 2 group, *N* = 81	IC = 3 group, *N* = 81	*p*-value	IC = 2 group, *N* = 81	IC = 3 group, *N* = 81	*p*-value	IC = 2 group, *N* = 81	IC = 3 group, *N* = 81	*p*-value	IC = 2 group, *N* = 81	IC = 3 group, *N* = 81	*p*-value
Clinica stage												
III	92.9	97.2	0.6	78.9	91.7	0.14	84.2	97.1	0.058	94.1	94.5	0.99
IVa	85.4	71.1	0.29	68.3	69.5	0.96	78.0	74.3	0.69	89.9	94.7	0.38
LDH level (U/L)												
< 245	88.7	87.1	0.46	79.2	82.8	0.66	86.5	87.1	0.96	92.1	95.5	0.43
≥ 245	80.8	39.8	0.18	40.0	57.7	0.41	48.6	66.3	0.35	90.9	88.9	0.82

*LDH* lactate dehydrogenase, *OS* overall survival, *PFS* progression-free survival, *DMFS* distant metastasis-free survival, *LRRFS* locoregional relapse-free survival

### Patients with N2-3 category

We further analyzed the efficacy and safety of two versus three cycles of IC in NPC patients with N2-3 category. After propensity score matching, each group contained 138 patients with N2-3 disease. Among them, the IC = 2 group included 48 patients with N2 category and 21 with N3 category; the IC = 3 group included 48 patients with N2 category and 21 with N3 category (see Supplementary Table 1 for detailed distribution). A flowchart of the patient population screening process (Supplementary Fig. 1) and the clinicopathologic characteristics of the two groups of patients before and after PSM (Supplementary Table 1) can be found in detail in the Supplementary Material. Long-term efficacy analysis showed no significant differences in 5-year OS, PFS, DMFS, or LRRFS rates between the IC = 2 and IC = 3 groups (Supplementary Fig. 2). Multivariate analysis revealed the N3 category as an independent prognostic factor for OS, while both the N3 category and LDH levels ≥ 245 U/L were independent predictors of worse DMFS and PFS (Supplementary Table 2). The IC = 3 group had a significantly higher incidence of grade 3/4 thrombocytopenia and anemia compared to the IC = 2 group (Supplementary Table 3). In these exploratory subgroup analyses, among patients with N2 category, three cycles of IC were associated with improved PFS and DMFS compared with two cycles; however, no interaction test was performed to confirm a differential treatment effect by N category. Conversely, for those with the N3 category, the number of IC cycles did not significantly affect clinical outcomes (Supplementary Table 4).

## Discussion

There is still controversy regarding the optimal IC cycle for LA-NPC patients. In this study, it was determined that administering two cycles of IC for LA-NPC was comparable to three cycles in terms of efficacy, while also reducing the incidence of treatment-related acute toxicity during CCRT. Additionally, subgroup analyses indicated that two cycles of IC confer a survival advantage similar to that of three cycles for patients in both high- and low-risk categories. These exploratory findings suggest that two cycles of IC therapy may be adequate for patients with LA-NPC, although the study was not powered to detect treatment effect modification in specific subgroups, and the results should not be regarded as definitive.

For patients with LA-NPC, CCRT has become the recommended treatment regimen, which combines the synergistic effects of chemotherapy and radiotherapy to improve treatment outcomes [9]. With the development of radiotherapy techniques, such as IMRT and volumetric modulated arc therapy (VMAT), the local control rate of NPC has increased significantly, and the main reason for patients’ treatment failure is the occurrence of distant metastases [28–30]. The addition of IC to CCRT in the treatment regimen of LA-nasopharyngeal carcinoma has been shown in previous studies to significantly improve the survival of patients [12, 13]. In randomized trials and clinical practice, the preference for IC with three cycles is often observed among physicians. Given that patients with LA-NPC may already have micrometastases at diagnosis and face a higher risk of disease progression [10], they may theoretically benefit from intensified treatment. Nevertheless, a considerable proportion of patients may be unable to complete the prescribed three cycles due to treatment-related toxicity and financial constraints. The optimal number of IC cycles for maximizing patient survival remains uncertain, necessitating further research to ascertain the most effective number of cycles. This research could help to balance the therapeutic benefits with the associated risks and costs, ultimately guiding personalized treatment strategies for improved patient outcomes.

Our study revealed that administering three cycles of IC did not improve OS, DMFS, LRRFS, or PFS in patients with LA-NPC, aligning with findings from previous research [31]. One plausible explanation is that two cycles of IC may suffice to eradicate micrometastases and diminish tumor burden in these patients. Additionally, the extension of IC to three cycles could potentially delay the initiation of radiotherapy, thereby increasing the interval between cancer diagnosis and the commencement of definitive radiotherapy. Prior studies have indicated that extended waiting periods for radiotherapy are linked to poorer prognoses across various cancers, including NPC [24, 32–34]. Patients receiving three cycles of IC generally experience longer delays before starting radiotherapy compared to those receiving only two cycles, which may negate the survival advantages of additional IC cycles. Consequently, the waiting time for radiotherapy emerges as a potential confounding factor that warrants careful consideration, as it may subtly influence the outcomes of this study.

In this study, we used multivariate Cox’s analysis to determine which variables affect the survival outcomes of patients, and based on this, we identified those patients who were at relatively high risk. The identification of stage IVa as an independent prognostic factor for OS, DMFS, and PFS in our multivariate analysis underscores the importance of disease stage in determining treatment outcomes. Similarly, the association of high LDH levels with adverse outcomes for DMFS and PFS is consistent with previous research, indicating that LDH may serve as a biomarker for disease aggressiveness and treatment resistance [35]. However, subgroup analyses revealed no statistically significant differences in prognostic outcomes between patients receiving two versus three IC cycles when stratified by clinical stage and LDH levels.

The determination of the optimal number of IC cycles for patients with LA-NPC remains a subject of debate. Evidence from a prior retrospective cohort study, which examined 247 matched pairs of patients with advanced NPC, indicated no significant difference in survival outcomes between those receiving two IC cycles and those receiving three to four IC cycles. However, in the subgroup analysis of stage N2-3 patients, patients treated with two IC cycles had a higher OS rate than those treated with three to four IC cycles (92.4% vs. 80.8%, *P* = 0.029) [19]. Another retrospective study analysis showed that two cycles of IC significantly improved DMFS and PFS in LA-NPC patients compared with three to four cycles of IC. Moreover, the IC cycle was an independent predictor of DMFS and PFS in patients in both univariate and multivariate analyses [36]. Nevertheless, the aforementioned investigations grouped patients receiving three or four cycles of IC into a single ‘high cycle’ category, neglecting to assess the impact of three cycles independently. It is a common clinical practice and a standard in randomized controlled trials to administer three cycles of IC. However, two cycles of IC have also been investigated in prospective randomized trials with favorable outcomes, lending support to the adequacy of this approach. For instance, the phase 3 trial by Lv et al. compared two cycles of lobaplatin and fluorouracil versus cisplatin and fluorouracil induction chemotherapy followed by concurrent chemoradiotherapy in patients with stage III–IVB NPC, and reported non-inferior 5-year progression-free survival (75.0% vs. 75.5%) with reduced toxicities [37]. Additionally, the randomized phase 3 trial by Li et al. demonstrated that two cycles of induction TPC (paclitaxel, cisplatin, and capecitabine) significantly improved failure-free survival compared with PF in stage IVA–IVB NPC (3-year FFS 83.5% vs. 68.9%) [38]. These prospective data indicate that two cycles of IC can achieve satisfactory disease control, and suggest that additional cycles might not be necessary. A retrospective analysis conducted in 2020 assessed the survival outcomes of patients undergoing two, three, or four cycles of IC. The study found no significant difference in survival rates between groups receiving two or three cycles of IC. In contrast, the group that received four cycles of IC exhibited a reduced OS rate and an increased prevalence of treatment-related toxicities [18]. However, this study included NPC patients with stage II, and IC is not routinely recommended for these patients according to the most recent guidelines [9]. Furthermore, the patient cohorts in these studies were classified using the 7th edition of the AJCC/UICC staging system, which may reduce the applicability of their findings in the context of modern clinical practice now that the 8th edition of the staging system has been introduced [26]. Jiang et al.‘s findings indicated that in NPC patients with stage III-IVa, who were included in the study based on the 8th AJCC/UICC staging system, 3 cycles of IC did not provide any additional survival benefit. However, in that study, patients had different induction chemotherapy regimens, which could be a potential confounding factor that would have biased the results somewhat. Patients who receive different induction chemotherapy regimens have varying survival prognoses and rates of treatment-related toxicity [39–41]. In contrast to the results of these four studies, another study indicated that NPC patients with stage N2-3 had improved survival with 4 cycles of IC compared to 2 cycles [42]. This study specifically examined patients with advanced stage N, excluding those who underwent three cycles of IC. Although the results of these studies differ, they suggest that adding more IC cycles might not improve survival outcomes, and our results support this. Unlike previous research, our study specifically examined NPC patients at stage III-IVa treated with TPF induction chemotherapy, following the 8th edition of the AJCC/UICC staging system.

Additionally, an important finding of our study is the increased incidence of grade 3/4 treatment-related acute toxicity in the three-cycles group, particularly during CCRT. This finding is consistent with the notion that while an intensified treatment regimen may enhance the cytotoxic effect on cancer cells, it also poses a higher risk of adverse effects [42]. The clinical implications of this finding are profound, as it suggests that the pursuit of improved survival outcomes through the escalation of IC cycles may come at the cost of increased toxicity, which can negatively impact patients’ quality of life and potentially their long-term health.

Our study used PSM to minimize selection bias and the comprehensive analysis of both survival outcomes and treatment-related toxicities. However, it is not without limitations. The retrospective nature of the study may introduce biases that cannot be fully accounted for. Additionally, the follow-up period may not be sufficient to capture potential late recurrences or differences in long-term survival. This constraint was influenced by the availability of the original dataset. Importantly, baseline plasma Epstein-Barr virus (EBV) DNA levels, a well-established prognostic and risk-stratification factor in nasopharyngeal carcinoma, were not recorded in the original dataset and could not be incorporated into the propensity score matching or multivariable models. In clinical practice, patients with higher EBV-DNA levels might be more likely to receive three cycles of induction chemotherapy, representing a potential selection bias. The absence of this variable could confound the comparison of survival outcomes and limit the validity of our findings. Notably, a critical confounder—the time interval from the end of induction chemotherapy to the initiation of radiotherapy—was not recorded in the original dataset and therefore could not be incorporated into the propensity score matching or multivariable adjustment models. Since three cycles of IC inherently prolong the time to radiotherapy compared with two cycles, this unmeasured variable may have confounded the survival comparisons and could potentially bias our results. This limitation should be carefully considered when interpreting the study findings. Additionally, although the induction chemotherapy was uniformly TPF-based, the concurrent chemotherapy phase involved different platinum agents (cisplatin or nedaplatin) and administration schedules (weekly or tri-weekly). While the chemotherapy regimen variable was included as a covariate and was balanced after propensity score matching, the specific scheduling differences were not explicitly adjusted for and could have influenced both toxicity and efficacy outcomes, representing another source of potential residual confounding. This limitation should be considered when interpreting the comparative results. Future studies with extended follow-up durations and detailed recording of timing variables would be valuable to confirm our findings regarding long-term outcomes. Furthermore, the subgroup analyses were exploratory and constrained by limited sample sizes, which precluded robust statistical inference; therefore, these results should not be interpreted as conclusive evidence of equivalence between the two IC cycles in any risk stratum. Additionally, detailed data on dose reductions, temporary interruptions, or cumulative delivered doses of concurrent chemotherapy were not captured in the original dataset, which precluded a more nuanced analysis of treatment intensity and its potential impact on survival outcomes. This limitation should be considered when interpreting the toxicity‑tolerance and efficacy comparisons. Moreover, the generalizability of our findings may be limited by the homogeneity of our patient population, which predominantly consists of LA-NPC patients from a single ethnic background. Additionally, no formal interaction tests were conducted in the subgroup analyses, and the limited sample sizes in certain strata preclude robust inference regarding differential treatment effects; these analyses should therefore be considered exploratory and hypothesis-generating only. Taken together, the findings of this study could assist patients in avoiding unnecessary treatment and reducing financial costs. Administering appropriate cycles of IC may enhance patient compliance with CCRT.

## Conclusion

In conclusion, in this retrospective study, three cycles of IC were not associated with a significant improvement in survival compared with two cycles, but three cycles of IC were associated with increased toxicity. These findings, together with the exploratory subgroup analyses, warrant cautious interpretation and further validation in prospective studies with adequate power for subgroup evaluations. As a result of these findings, a balanced approach to treatment planning is needed, taking potential risks and benefits into consideration. These findings warrant future prospective, multicenter studies to confirm them and explore the factors that could affect treatment response and toxicity. 

## Supplementary Information

Supplementary Material 1: Supplementary Figure 1. Flow-chart of the patient selection procedure. Supplementary Figure 2. Kaplan-Meier survival curves of the IC=2 group and IC=3 group in patients with N2-3 category after matching. A. Overall survival; B. Progression-free survival; C. Distant metastasis-free survival; D. Locoregional-free survival. Supplementary Table 1. Patient demographics and baseline characteristics. Supplementary Table 2. Multivariate analyses of the OS, PFS, DMFS and LRRFS rate by Cox proportional hazards regression models after PSM. Supplementary Table 3. Incidence of grade 3 acute toxicities during treatment in patients with N2-3 category after PSM. Supplementary Table 4. Subgroup analysis.

## Data Availability

The datasets used and/or analysed during the current study are available from the corresponding author on reasonable request.

## Abbreviations

IC: Induction Chemotherapy
CCRT: Concurrent Chemoradiotherapy
LA-NPC: Locally Advanced Nasopharyngeal Carcinoma
CT: Computed Tomography
MRI: Magnetic Resonance Imaging
OS: Overall Survival
PFS: Progression-Free Survival
DMFS: Distant Metastasis-Free Survival
LRRFS: Locoregional Relapse-Free Survival
PSM: Propensity Score Matching
IMRT: Intensity-Modulated Radiotherapy
EBV: Epstein-Barr Virus
ECOG: Eastern Cooperative Oncology Group
CTCAE: Common Terminology Criteria for Adverse Events
GTV: Gross Tumor Volume
CTV: Clinical Target Volume
PTV: Planning Target Volume
LDH: Lactate Dehydrogenase
BMI: Body Mass Index
TPF: Docetaxel, Cisplatin, 5-Fluorouracil
TNF: Docetaxel, Nedaplatin, 5-Fluorouracil
AJCC: American Joint Committee on Cancer
UICC: Union for International Cancer Control
WHO: World Health Organization
aHR: Adjusted Hazard Ratio
CI: Confidence Interval
VMAT: Volumetric Modulated Arc Therapy
