# Supplementary material for: Comparative efficacy and toxicity of two versus three induction chemotherapy cycles in locally advanced nasopharyngeal carcinoma: a propensity score matching analysis
Source: BMC Cancer. 2026 Jul 31;26:1322. doi: 10.1186/s12885-026-16655-2 (PMC13721493; doi:10.1186/s12885-026-16655-2)
Supplement: Supplementary file 1 [file 12885_2026_16655_MOESM1_ESM.docx]

**Supplementary materials**

**Supplementary Figure 1**. Flow-chart of the patient selection procedure.

**Supplementary Figure 2.** Kaplan-Meier survival curves of the IC=2 group and IC=3 group in patients with N2-3 category after matching. A. Overall survival; B. Progression-free survival; C. Distant metastasis-free survival; D. Locoregional-free survival.

**Supplementary Table 1.** Patient demographics and baseline characteristics.

**Supplementary Table 2.** Multivariate analyses of the OS, PFS, DMFS and LRRFS rate by Cox proportional hazards regression models after PSM.

**Supplementary Table 3.** Incidence of grade 3 acute toxicities during treatment in patients with N2-3 category after PSM.

**Supplementary Table 4.** Subgroup analysis.

**Supplementary Figure 1**. Flow-chart of the patient selection procedure.

**Supplementary Figure 2.** Kaplan-Meier survival curves of the IC=2 group and IC=3 group in patients with N2-3 category after matching. A. Overall survival; B. Progression-free survival; C. Distant metastasis-free survival; D. Locoregional-free survival.

**Table1.** Patient demographics and baseline characteristics

| **Characteristics** | **Before matching** | | | **After matching** | | |
| --- | --- | --- | --- | --- | --- | --- |
|  | **IC=2 group, N = 90** | **IC=3 group, N = 69** | **p-value** | **IC=2 group, N = 69** | **IC=3 group, N = 69** | **p-value** |
| **Age (Mean ± SD)** | 46 ± 10 | 47 ± 11 | 0.648 | 46 ± 11 | 47 ± 11 | 0.601 |
| **Gender** |  |  | 0.721 |  |  | 0.830 |
| Male, n (%) | 75 (83%) | 56 (81%) |  | 55 (80%) | 56 (81%) |  |
| Female, n (%) | 15 (17%) | 13 (19%) |  | 14 (20%) | 13 (19%) |  |
| **Histology** |  |  | >0.999 |  |  | >0.999 |
| WHO II | 2 (2%) | 2 (3%) |  | 2 (3%) | 2 (3%) |  |
| WHO III | 88 (98%) | 67 (97%) |  | 67 (97%) | 67 (97%) |  |
| **T stage** |  |  | 0.038* |  |  | 0.586 |
| T1-2 | 42 (47%) | 21 (30%) |  | 24 (35%) | 21 (30%) |  |
| T3-4 | 48 (53%) | 48 (70%) |  | 45 (65%) | 48 (70%) |  |
| **N stage** |  |  | 0.601 |  |  | >0.999 |
| N2 | 66 (73%) | 48 (70%) |  | 48 (70%) | 48 (70%) |  |
| N3 | 24 (27%) | 21 (30%) |  | 21 (30%) | 21 (30%) |  |
| **Clinica stage** |  |  | 0.059 |  |  | 0.394 |
| III | 54 (60%) | 31 (45%) |  | 36 (52%) | 31 (45%) |  |
| IVa | 36 (40%) | 38 (55%) |  | 33 (48%) | 38 (55%) |  |
| **Smoking** |  |  | 0.191 |  |  | 0.488 |
| No | 60 (67%) | 39 (57%) |  | 43 (62%) | 39 (57%) |  |
| Yes | 30 (33%) | 30 (43%) |  | 26 (38%) | 30 (43%) |  |
| **BMI** |  |  | 0.467 |  |  | >0.999 |
| < 18.5 | 6 (7%) | 2 (3%) |  | 1 (1%) | 2 (3%) |  |
| ≥ 18.5 | 84 (93%) | 67 (97%) |  | 68 (99%) | 67 (97%) |  |
| **LDH level (U/L)** |  |  | 0.501 |  |  | 0.813 |
| < 245 | 79 (88%) | 58 (84%) |  | 59 (86%) | 58 (84%) |  |
| ≥ 245 | 11 (12%) | 11 (16%) |  | 10 (14%) | 11 (16%) |  |
| **Platinum dose of CCRT (mg/m2)** |  |  | 0.004* |  |  | 0.120 |
| < 200 | 38 (42%) | 45 (65%) |  | 36 (52%) | 45 (65%) |  |
| ≥ 200 | 52 (58%) | 24 (35%) |  | 33 (48%) | 24 (35%) |  |
| **Chemotherapy regimen** |  |  | 0.004* |  |  | >0.999 |
| TPF + P | 38 (42%) | 45 (65%) |  | 33 (48%) | 33 (48%) |  |
| TNF + N | 52 (58%) | 24 (35%) |  | 36 (52%) | 36 (52%) |  |

IC, induction chemotherapy; CCRT, concurrent chemoradiotherapy; BMI, body mass index; LDH, lactate dehydrogenase; TPF + P, docetaxel plus nedaplatin and 5-fluorouracil followed by nedaplatin-based CCRT; TNF + N, docetaxel plus cisplatin and 5-fluorouracil followed by cisplatin-based CCRT ; PSM, propensity score-matched.

**Table2.** Multivariate analyses of the OS, PFS, DMFS and LRRFS rate by Cox proportional hazards regression models after PSM

|  | **OS** | | **PFS** | | **DMFS** | | **LRRFS** | |
| --- | --- | --- | --- | --- | --- | --- | --- | --- |
|  | **aHR(95% CI)** | **p-value** | **aHR(95%CI)** | **p-value** | **aHR(95%CI)** | **p-value** | **aHR(95%CI)** | **p-value** |
| Gender (Male vs. Female) | N/A | N/A | 0.41 (0.12~1.35) | 0.143 | N/A | N/A | N/A | N/A |
| N stage (N2 vs. N3) | 6.23 (2.18~17.76) | 0.001* | 2.92 (1.5~5.68) | 0.002* | 3.02 (1.42~6.41) | 0.004* | N/A | N/A |
| Clinical stage (III vs. IVa) | N/A | N/A | N/A | N/A | N/A | N/A | 2.24 (0.48~10.39) | 0.305 |
| LDH (< 275 vs. ≥ 275) | 1.21 (0.39~3.77) | 0.739 | 2.54 (1.19~5.41) | 0.016* | 2.65 (1.11~6.31) | 0.028* | 1.93 (0.36~10.41) | 0.444 |
| IC cycle (2 vs. 3) | 2.43 (0.87~6.78) | 0.089 | 0.65 (0.32~1.3) | 0.218 | 0.76 (0.35~1.64) | 0.48 | 0.33 (0.06~1.8) | 0.2 |
| Platinum dose (< 200 vs. ≥ 200) | 1.83 (0.65~5.09) | 0.251 | 1.47 (0.73~2.97) | 0.283 | 1.28 (0.57~2.85) | 0.549 | 2.97 (0.55~16.03) | 0.206 |

PSM, propensity score-matched; OS, overall survival; PFS, progression-free survival; DMFS, distant metastasis-free survival; LRRFS, locoregional relapse-free survival; N/A, not applicable.

**Table 3.** Incidence of grade 3 acute toxicities during treatment in patients after PSM

| Acute Toxicities | **During treatment** | | | **During IC** | | | **During CCRT** | | |
| --- | --- | --- | --- | --- | --- | --- | --- | --- | --- |
|  | **IC=2 group, N = 69** | **IC=3 group, N = 69** | **p-value** | **IC=2 group, N = 69** | **IC=3 group, N = 69** | **p-value** | **IC=2 group, N = 69** | **IC=3 group, N = 69** | **p-value** |
| **Haematologic** |  |  |  |  |  |  |  |  |  |
| Leucopenia | 26 (37.7) | 23 (33.3) | 0.594 | 6 (8.7) | 6 (8.7) | 1 | 26 (37.7) | 19 (27.5) | 0.204 |
| Neutropenia | 23 (33.3) | 21 (30.4) | 0.715 | 14 (20.3) | 14 (20.3) | 1 | 22 (31.9) | 13 (18.8) | 0.078 |
| Anaemia | 3 (4.3) | 10 (14.5) | 0.041* | 1 (1.4) | 0 (0) | 1 | 4 (5.8) | 11 (15.9) | 0.056 |
| Thrombocytopenia | 5 (7.2) | 16 (23.2) | 0.009* | 1 (1.4) | 0 (0) | 1 | 6 (8.7) | 11 (15.9) | 0.195 |
| **Non-Haematologic** |  |  |  |  |  |  |  |  |  |
| Vomiting | 5 (7.2) | 9 (13) | 0.259 | 3 (4.3) | 5 (7.2) | 0.718 | 5 (7.2) | 9 (13) | 0.259 |
| Weight loss | 2 (2.9) | 2 (2.9) | 1 | 1 (1.4) | 2 (2.9) | 1 | 2 (2.9) | 2 (2.9) | 1 |
| Hypoalbuminemia | 0 (0) | 0 (0) | 1 | 0 (0) | 0 (0) | 1 | 0 (0) | 0 (0) | 1 |
| Liver/Kidney damage | 0 (0) | 0 (0) | 1 | 0 (0) | 0 (0) | 1 | 0 (0) | 0 (0) | 1 |

PSM, propensity score-matched; IC, induction chemotherapy; CCRT, concurrent chemoradiotherapy; N/A, not applicable.

**Table 4.** Subgroup analysis

| Characteristic | **OS (%)** | | | **PFS (%)** | | | **DMFS (%)** | | | **LRRFS (%)** | | |
| --- | --- | --- | --- | --- | --- | --- | --- | --- | --- | --- | --- | --- |
|  | **IC=2 group, N = 81** | **IC=3 group, N = 81** | **p-value** | **IC=2 group, N = 81** | **IC=3 group, N = 81** | **p-value** | **IC=2 group, N = 81** | **IC=3 group, N = 81** | **p-value** | **IC=2 group, N = 81** | **IC=3 group, N = 81** | **p-value** |
| **N stage** |  |  |  |  |  |  |  |  |  |  |  |  |
| N2 | 89.1 | 90.4 | 0.30 | 70.1 | 91.5 | 0.023* | 74.1 | 95.7 | 0.012* | 95.4 | 95.7 | 0.95 |
| N3 | 80.7 | 54.6 | 0.17 | 59.9 | 49.2 | 0.39 | 74.7 | 49.2 | 0.11 | 84.2 | 100.0 | 0.10 |
| **LDH level (U/L)** |  |  |  |  |  |  |  |  |  |  |  |  |
| < 245 | 85.7 | 85.0 | 0.47 | 75.4 | 82.7 | 0.41 | 82.3 | 84.4 | 0.84 | 92.6 | 98.1 | 0.16 |
| ≥ 245 | 85.7 | 39.8 | 0.044* | 30.0 | 57.7 | 0.34 | 40.0 | 66.3 | 0.32 | 88.9 | 88.9 | 0.93 |

LDH, lactate dehydrogenase; OS, overall survival; PFS, progression-free survival; DMFS, distant metastasis-free survival; LRRFS, locoregional relapse-free survival
